# From Theory to Practice: A Methodology Article Describing the Eight-Stage Health Education Planning Instrument in Community Health Education

**DOI:** 10.3390/healthcare14040436

**Published:** 2026-02-09

**Authors:** Carla Camí, Teresa Botigué, Judith Roca, Rosa Mar Alzuria-Alós, Elena Paraíso-Pueyo, Laia Selva-Pareja

**Affiliations:** 1 Department of Nursing and Physiotherapy, University of Lleida, 25198 Lleida, Spain; 2 Health Education, Nursing, Sustainability and Innovation Research Group (GREISI), 25198 Lleida, Spain; 3 Lleida Institute for Biomedical Research, Dr. Pifarré Foundation, IRB Lleida, 25198 Lleida, Spain; 4 Chair of Development of Health and Sustainability Organizations and Territories (DOTSS), University of Lleida, 25001 Lleida, Spain

**Keywords:** community preparedness, COVID-19, emotions, hand hygiene, health behavior, health education, health knowledge, attitudes, practice, respiratory infection prevention

## Abstract

**Highlights:**

**What are the main findings?**

- The Eight-Stage Health Education Planning Instrument is described and applied for the first time within a scientific framework through a community-based COVID-19 prevention intervention.
- This instrument proves useful for improving population preparedness against respiratory health threats beyond COVID-19.

**What are the implications of the main findings?**

- The study provides a clear example of how the Eight-Stage Instrument can be applied in local community interventions.
- It offers practical guidance for future adaptation, implementation, and evaluation of public health behavior change initiatives.

**Abstract:**

**Background/Objectives:** This methodology article describes the development and application of a structured planning instrument for community health education interventions. Community health education can strengthen population preparedness against respiratory threats beyond COVID-19 by improving knowledge, attitudes, and practices. The aim was to describe the development and implementation of the Eight-Stage Health Education Planning Instrument as applied in a community health education intervention aimed at improving preventive behaviors against COVID-19. **Methods**: A systematic, theory-based, and practice-oriented process was followed. Planning was guided by an Eight-Stage Health Education Planning Instrument. Conceptual models (ASE model, TTM, and I-Change model) were used to identify target determinants, and the Behavior Change Wheel guided the mapping stage from determinants to intervention functions and techniques. **Results**: The community health education intervention, developed using the Eight-Stage Health Education Planning Instrument, was named “Rethinking COVID-19” and consisted of a two-hour, in-person workshop combining brief didactic content with interactive components across three blocks: hand hygiene, healthy lifestyle, and emotional coping. Delivery procedures were predefined to ensure fidelity. The first two workshops served as formative pilots, allowing for the refinement of materials and procedures. Adjustments were guided by facilitator observations, immediate debriefs, participant satisfaction data, and one-word feedback descriptors. **Conclusions**: This methodology article presents the Eight-Stage Health Education Planning Instrument, a structured yet adaptable framework for the design, implementation, and evaluation of community health education programs. Its systematic stages provide an effective means of integrating theory, evidence, and community context. The “Rethinking COVID-19” intervention illustrates how the instrument standardizes planning while remaining responsive to local needs and emerging challenges. By incorporating behavioral models and clearly distinguishing between evaluation and follow-up, it strengthens methodological rigor and supports institutionalization.

## 1. Introduction

The COVID-19 pandemic was an unprecedented situation that triggered a severe public health crisis worldwide [1]. Thus, on 30 January 2020, the World Health Organization (WHO) declared the virus outbreak as an international public health emergency [2], underlining its severity and the need for a coordinated global response. Aside from the worldwide health threat, the pandemic had significant consequences in different areas [3], such as social-, economic-, environmental-, and energy-related ones [4]. In line with the evolution of the post-pandemic context, it is evident that efforts should be directed toward community preparedness against respiratory threats and health literacy as cross-cutting capacities beyond COVID-19.

Faced with these challenges, community health education (HE) interventions became established as essential strategies for improving the well-being and quality of life of individuals [5,6]. It must be noted that HE increases health-related knowledge, promotes healthy lifestyles [7], and addresses motivational factors that drive the population to adopt healthy behaviors [8]. Thus, in the context of the pandemic, HE has been fundamental for empowering the population to make informed decisions about their well-being. Through health literacy, people acquire the necessary skills to better understand and critically assess the health information available, which facilitates informed decision making. This literacy translates into a greater capacity for protection and response against critical situations, such as those experienced during the COVID-19 pandemic [9].

To design any HE intervention, it is necessary to perform an analysis of the current reality, which allows for the identification of the current needs to be included and addressed and the context of the intervention population to be identified [6]. In this regard, a previous mixed-methods study was conducted to examine the situation in the Segrià region (Lleida, Spain) [10]. The study assessed community HE needs in a community sample of 1559 participants (quantitative component) and 19 participants (qualitative component) in January and February, 2022. The results showed important gaps in knowledge regarding the correct handwashing technique, despite a general awareness of COVID-19 preventive measures. In addition, difficulties in managing the psychological impact of the pandemic persisted in a substantial proportion of participants, with 69.5% reporting a psychological impact after two years. Based on these findings, the authors concluded that a community HE intervention was needed to address the identified knowledge gaps and psychological challenges related to preventive behaviors against COVID-19.

Need-based and territory-oriented approaches have been widely recognized as essential for guiding the design and allocation of community health interventions, particularly in rural and community settings. Recent mixed-methods research highlights the value of systematic need assessment as a foundation for context-sensitive planning and resource prioritization in community health programs [11].

For an intervention to be effective and respond to these needs, the use of conceptual models and theories to guide its design is essential [12]. One of the models that can be applied to HE is the Attitude, Social influence, and Efficacy (ASE) model, along with the Transtheoretical Model (TTM) and the I-Change model. These models support the understanding of factors that influence health at both individual and social levels, thereby enabling the design of more effective interventions adapted to the context [12]. Specifically, the ASE model seeks to understand how people make health decisions, considering three key variables: the attitude towards a behavior or conduct, the social influence, and self-efficacy [13,14]. On its part, the TTM describes the process of behavioral change through six stages: precontemplation, contemplation, preparation, action, maintenance, and termination [15,16]. Lastly, the I-Change model establishes that the intention and skills of a person are fundamental for adopting healthy behaviors, with these being influenced by motivation, and with motivation dependent on the ASE model [17].

Following the taxonomy proposed by Selva-Pareja [12], a distinction is made between (a) logical “instruments” or implementation planning, which structure the process, and (b) conceptual models and theories, which explain the determinants that should be addressed. Whereas conceptual models and theories explain why and what should be targeted to achieve behavior change, logical “instruments” specify how to plan and organize the intervention in practice.

Despite the availability of established methodological frameworks and reporting guidelines in health research, the meta-epidemiological evidence indicates that the application of complex methodological tools may be inconsistent in the absence of clear operational guidance. In particular, a lack of structured and contextualized instructions has been shown to limit the reliability, coherence, and interpretability of methodological assessment processes, even when conducted by highly experienced professionals [18]. These findings highlight the need for structured, theory-informed, and methodologically transparent planning approaches, particularly in complex community HE interventions, where translating theory into practice remains a key challenge.

In this regard, the aim of this methodology article was to describe the development and implementation of the Eight-Stage Health Education Planning Instrument applied in a community HE intervention to improve the participants’ knowledge, attitudes, and practices (KAP) regarding preventive behaviors against COVID-19, in response to previously identified needs [10].

## 2. Materials and Methods

Given the methodological nature of this study, Section 2 focuses on describing the structure and logic of the instrument, whereas Section 3 demonstrates its practical application.

This approach is based on the principles of knowledge translation in community health settings, which emphasize systematically applying situational and theoretical evidence to support effective, context-sensitive interventions [19,20].

### 2.1. Overview of the Process

A systematic, theory-based, and practice-oriented process was followed to develop a community HE intervention addressing KAP related to COVID-19 preventive behaviors in the Segrià region.

**Figure 1:**
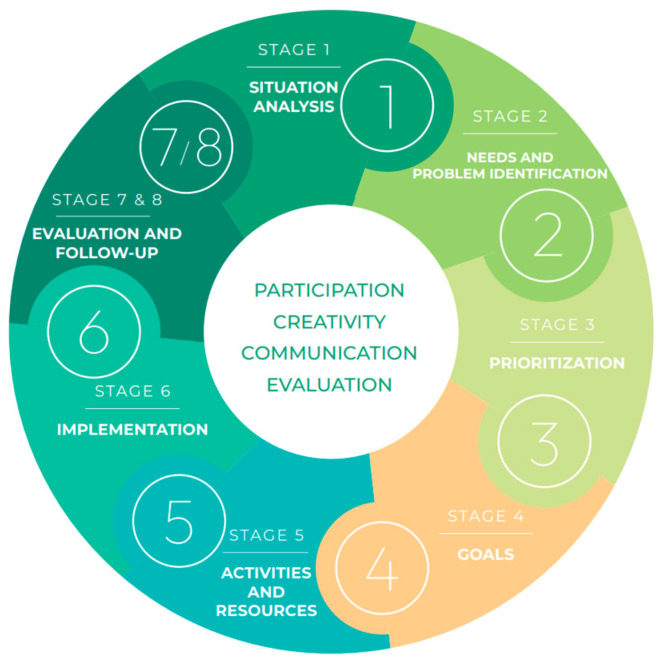
Operational structure of the Eight-Stage Health Education Planning Instrument for community HE interventions. Source: adapted from Gómez et al. [6].

An operational instrument for planning HE interventions was guided by the Eight-Stage Health Education Planning Instrument, which serves as a logical model for planning [6] (Figure 1). The instrument standardizes decision points and deliverables at each stage, separates evaluation from follow-up to institutionalized sustainability checks, and supports flexible points of entry to reflect real-world constraints.

In this article, a detailed description and application of the Eight-Stage Health Education Planning Instrument is presented for the first time. This approach was conceptually aligned with established frameworks, predisposing, reinforcing and enabling causes in educational diagnosis and evaluation (PRECEDE) and policy, regulatory, organizational, educational, environmental and development (PROCEED) [21,22,23], and Intervention Mapping [24] (Table 1).

### 2.2. The Eight Stages of the Health Education Planning Instrument

This section introduces the Eight-Stage Health Education Planning Instrument as a logical and operational framework for planning HE interventions. The stages can guide professionals from problem identification to sustainability assessment, ensuring the systematic integration of theory, evidence, and community context. The eight stages of the instrument are as follows:

1. Stage 1. Situation analysis: needs assessment to identify priority health issues and target populations.
  The situation analysis represents the foundational stage for any HE program. It involves systematically collecting and interpreting information on the community’s sociodemographic profile, health status, and available resources to identify priority health problems and guide subsequent stages of planning.
2. Stage 2. Needs and problem identification.
  This stage involves analyzing and interpreting data gathered during the situation analysis stage to identify the community’s perceived, expressed, and normative needs, as well as available health assets. The goal is to understand the root causes of health problems and determine which behaviors or determinants require intervention.
3. Stage 3. Prioritization: prioritization of behaviors, identification of sub-behaviors, and selection of relevant theories or models.
  This stage focuses on ranking and selecting the most relevant health problems or behaviors to address and alignment with available resources and community priorities. It involves identifying specific sub-behaviors and the psychosocial determinants underlying them, as well as choosing the theoretical models that best explain and guide change.
4. Stage 4. Goals and measurable objectives: goal and objective setting, defining measurable and attainable outcomes.
  This stage translates the prioritized health problems and behaviors into clear program goals and measurable objectives. Goals describe the desired long-term impact of the intervention, while objectives specify short- and medium-term outcomes that are observable and quantifiable. Objectives should be Specific, Measurable, Achievable, Relevant, and Time-bound (SMART) and linked to behavioral and learning outcomes derived from the theoretical frameworks selected in the previous stage. Establishing these objectives provides the basis for designing intervention strategies, selecting indicators, and developing the evaluation plan.
5. Stage 5. Strategy selection, activities and resources: strategy formulation, aligning theoretical frameworks and intervention approaches; design of intervention activities, specifying actions and delivery formats.
  This stage focuses on translating goals and objectives into practical strategies and activities. It involves aligning the selected theoretical models with appropriate methods for behavior change, identifying the most effective intervention functions and defining the sequence and format of the activities. Resource planning is also addressed at this stage, including human, material, and temporal resources required for implementation. The deliverables include a comprehensive intervention plan describing strategies, core activities, supporting materials, and logistical requirements, ensuring that the intervention remains feasible, theory-based, and context-sensitive.
6. Stage 6. Implementation: implementation planning, mode of delivery, timing, and stakeholder involvement; optional: formative piloting and modifications.
  This stage operationalizes the planned intervention through structured implementation procedures. It defines the delivery mode, sequence, duration, and logistical coordination required to ensure fidelity and feasibility. Key elements include stakeholder engagement, facilitator training, scheduling, and resource management. A monitoring system is established to safeguard adherence to core components, while allowing for minor contextual adaptations. When applicable, formative piloting is conducted to test usability, acceptability, and flow, leading to iterative refinements before full-scale rollout.
7. Stages 7 and 8. Evaluation and follow-up: evaluation design, establishing indicators, methods, and timelines; follow-up planning, addressing continuity, feedback loops, and community engagement; and sustainability and institutionalization, ensuring the long-term integration of effective practices.
  The final stages combine a systematic evaluation and follow-up to ensure the quality, relevance, and sustainability of the interventions. The evaluation is designed across three dimensions—formative (before), process (during), and impact (after)—to examine suitability, fidelity, and achievement of objectives.
  Indicators are defined according to each level: participant reach and characteristics, satisfaction and acceptability, fidelity of delivery, and attainment of immediate outcomes. Data sources typically include attendance records, structured observations, facilitator checklists, and participant questionnaires.
  The follow-up focuses on maintaining engagement with community stakeholders, establishing mechanisms for feedback, and identifying opportunities for institutionalization of effective practices.

Each stage concludes with specific deliverables and decision checkpoints, promoting consistency and adaptability across intervention contexts (see Figure 1).

The following section illustrates the application of the instrument through a community HE intervention focused on COVID-19 prevention in the Segrià region, named “Rethinking COVID-19”.

## 3. Results

The design and delivery of a community HE intervention, named “Rethinking COVID-19” and structured using the Eight-Stage Health Education Planning Instrument, are presented below. To facilitate replication, the step-by-step application has been summarized, and a complete Template for Intervention Description and Replication (TIDieR) checklist [25] is provided in Appendix A.

### 3.1. Stage 1: Situation Analysis

In the present study, this stage was implemented through the study entitled “Health Education: an educational tool to prevent and deal with COVID-19”, which was part of a broader multidisciplinary project named “Science-based education and communication to fight COVID-19 and future pandemics (IlerCOVID)”.

#### Stage 1.1: Subjects and Sample Size Calculation

The study population consisted of individuals aged 18 years and older from various municipalities within the Segrià region. Participants were recruited through local city halls, and participation was completely voluntary. The intervention took place across all 38 municipalities in the region.

According to data from Idescat, dated 1 January 2021, the total population across these municipalities comprised 211,609 individuals, of whom 81.8% were aged 18 years or older (173,010 individuals). The sample size for the intervention was calculated based on the number of individuals aged 18 and above in the Segrià region, assuming an alpha error of 0.05, a statistical power of 0.8, a two-tailed comparison, and an anticipated effect size in the intervention group (Cohen’s d) of 0.5. Based on these parameters, the sample size for the intervention group was determined to be 128 participants. To enable a group comparison, an additional 128 participants were recruited as the control group. As no follow-up was conducted, participant attrition was not expected. Finally, the number of participants in each workshop was determined according to the room capacity provided by the city halls of each municipality.

### 3.2. Stage 2: Needs and Problem Identification

In the IlerCOVID project, the identification of needs was based on a previous mixed-methods study that combined surveys and focus groups conducted in the Segrià region to explore community knowledge, behaviors, and perceptions related to COVID-19 prevention and well-being [10].

### 3.3. Stage 3: Prioritization

As noted in Stage 2, the content of the community HE intervention “Rethinking COVID-19” was based on previously identified health needs [10]: (i) limited knowledge and practice regarding the correct handwashing technique, (ii) difficulties in managing the psychological impact after two years of the pandemic, and (iii) the need to reinforce healthy lifestyle habits despite generally positive self-reports.

Then, three behavioral domains were specified:

- Hand hygiene: correct technique at key moments.
- Healthy lifestyle: maintaining or enhancing physical activity and routines aligned with a Mediterranean-style diet.
- Emotional coping: recognizing pandemic-related emotions and applying simple self-regulation strategies.

Key barriers were identified across three domains:

- Hand hygiene: low familiarity with the correct sequence; perceived inconvenience; and lack of importance of “everyday” risk.
- Lifestyle: motivation fluctuates; misconceptions; and competing demands.
- Emotions: avoidance of difficult feelings; limited repertoire of coping skills; and stigma about asking for help.

Facilitators included high perceived usefulness of practical demonstrations, peer discussion, and take-home materials.

#### 3.3.1. Stage 3.1: Theoretical Underpinnings and Translation into Intervention Functions

**Figure 2:**
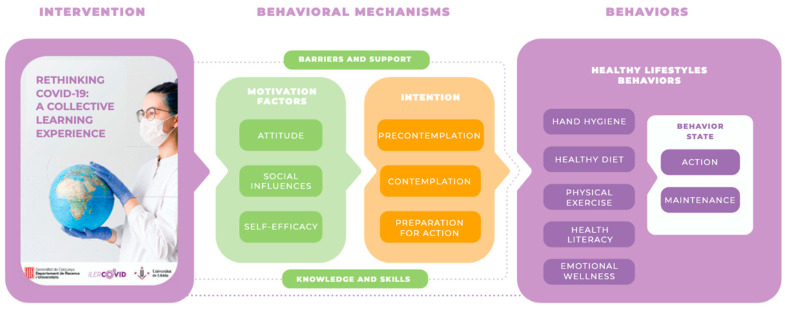
Pathway for developing the intervention “Rethinking COVID-19” according to the I-Change model.

The “Rethinking COVID-19” intervention was grounded on behavioral models. Following the methodology used by Homs et al. [26], a pathway was designed using the I-Change model, which integrates both the ASE model and the TTM. This integrated framework facilitated the development of the intervention and its alignment with behavioral mechanisms (Figure 2).

To identify behavioral determinants, three conceptual models were applied: the ASE model, the TTM, and the I-Change model. The ASE model was used to structure determinant mapping [12,13,14]; the TTM helped tailor messages and activities to the participants’ stage of change [15,16]; and the I-Change model integrated motivation (intention) with self-regulation processes such as risk perception, knowledge, and social support [17]. In this application, the ASE/I-Change constructs were used from Stage 2 onwards as an interpretive lens to classify needs into behavioral determinants, which were then prioritized and translated into objectives and activities (Stages 3–5).

Additionally, the Behavior Change Wheel (BCW) [27] served as a design tool to link these determinants to appropriate intervention functions during the activity selection phase. The TTM and BCW were used to inform stage-matched content and the selection of intervention functions during design (Stages 3–5) and were not applied as implementation or evaluation frameworks (i.e., participants’ stages of change were not assessed).

**Figure 3:**
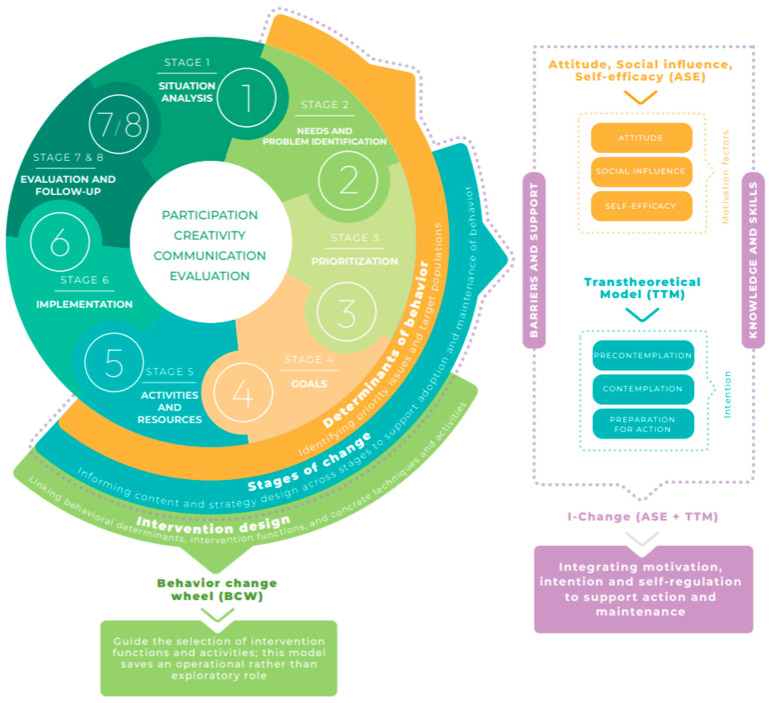
Application of the Eight-Stage Health Education Planning Instrument in the “Rethinking COVID-19” intervention. The figure illustrates how cross-cutting principles, planning stages, and selected behavioral models (ASE, Transtheoretical Model, and I-Change model) and the BCW were integrated in this specific application.

Figure 3 summarizes how the Eight-Stage Health Education Planning Instrument was operationalized in the “Rethinking COVID-19” intervention, illustrating the specific selection and integration of behavioral models and design frameworks used in this application.

The choice of theoretical models and their mapping to planning stages reflects the specific behaviors, determinants, and contextual needs addressed in this intervention and does not imply a prescriptive configuration for other applications.

#### 3.3.2. Stage 3.2: Practical Applications and Behavior Change Methods

The approach was operationalized through low-cost, group-based methods. Participants completed a guided practice with feedback activity, using paint to simulate soap in the handwashing demonstration and receiving corrections from peers. Barriers were elicited and re-attributed through structured group discussion focused on workable solutions. Misconceptions were addressed via myth-busting with immediate evidence in the “truth or myth” activity covering physical activity, diet, and sleep and media literacy. Simple action planning then prompted micro-plans for maintenance (when, where, and how). Finally, emotion identification and coping drills were conducted using the “box of emotions”, brief paced-breathing practice, and basic support mapping. Across all components, applications were designed to be stage-matched (TTM) and to build self-efficacy (ASE model) via mastery experiences and social persuasion.

### 3.4. Stage 4: Goals and Measurable Objectives

The program goal was to provide the adult population of Segrià with preventive and self-management strategies applicable to COVID-19 and future respiratory threats.

The overall objective was to increase the adoption of prioritized preventive behaviors (hand hygiene, basic healthy habits, and emotional coping) after the 2 h workshop, measured through process indicators and immediate outcomes.

Intermediate objectives were structured as follows:

- By the end of the workshop, ≥80% of participants will correctly order the six grouped steps of the handwashing technique (post-workshop questionnaire).
- By the end of the workshop, ≥90% will correctly identify four out of five statements related to preventive healthy habits and overall health (post-workshop questionnaire).
- By the end of the workshop, ≥80% will identify at least one coping strategy they can practice and recognize that emotions are not inherently positive or negative (post-workshop questionnaire).

Specific objectives corresponding to the activities were:

- During the workshop, ≥90% of participants will correctly perform the sequence of hand hygiene steps, verified through observation during the activity using gloves and paint.
- During the workshop, ≥80% of participants will recognize at least two strategies for maintaining healthy habits related to prevention and overall health.
- During the workshop, ≥80% of participants will identify ≥ two predominant emotions related to the pandemic.

It should be emphasized that, in this intervention, the intermediate objectives do not address behavioral change, since no medium- or long-term follow-up or evaluation was conducted.

### 3.5. Stage 5: Strategy Selection, Activities and Resources

The “Rethinking COVID-19” intervention focused on enhancing participants’ KAP concerning COVID-19 preventive behaviors.

#### 3.5.1. Stage 5.1: Design Principles to Instantiate Change Methods

The design drew on experiential learning (moving from demonstration to practice to feedback) while fostering social learning and peer discussion through modeling, attention to social norms, and relatedness. The materials and facilitation scripts were written in plain language to support a low literacy, with clear visuals and stepwise instructions. Prominence and personal relevance were enhanced by using everyday scenarios and local examples. Finally, self-regulation was scaffolded through simple planning prompts, cues, and basic support mapping. The materials included facilitator scripts, slides, the handwashing checklist from the Catalan Health Service [28], truth-or-myth prompts, emotion cards and envelopes, and a take-home brochure with key messages and resources.

#### 3.5.2. Stage 5.2: Activities

In accordance with the pathway developed and the identified needs, each session of “Rethinking COVID-19” was structured into three activities. Each activity included all phases of the TTM, considering motivational factors and potential barriers identified by the ASE model:

Activity 1. Hand hygiene: “Clean hands, lives saved”.

- Precontemplation: brief presentation about the importance of hand hygiene.
- Contemplation: discussion about the reasons why some individuals do not wash their hands frequently.
- Preparation: demonstration of the adequate handwashing technique using paint to simulate soap.
- Action: visualization of everyday situations that require handwashing.
- Maintenance: establishment of a personalized plan to maintain handwashing.

Activity 2. Healthy habits and COVID-19: Truths and myths about lifestyle habits and COVID-19.

- Precontemplation: introduction of the importance of healthy habits and the prevention of COVID-19.
- Contemplation: group discussion about the barriers against adopting healthy habits and prevention measures.
- Preparation: execution of the “truth or myth” activity. After each response, the correct answer was revealed and explained using scientific evidence to promote informed understanding.
- Action: presentation of specific strategies for healthy habits and the prevention of COVID-19.
- Maintenance: planning specific, long-term strategies.

Activity 3. Emotions: Box of emotions.

- Precontemplation and contemplation: individual reflection activity to identify the emotions experienced during the pandemic.
- Preparation and action: presentation of management of emotions strategies, such as deep breathing.

#### 3.5.3. Stage 5.3: Materials

The workshop used simple, low-cost materials aligned with each activity. For the guided hand-hygiene practice, each participant received single-use gloves; facilitators applied a small amount of paint to simulate soap, making coverage errors visible during the drill. To support the correct technique, printed hand-hygiene images/checklist from the Catalan Health Service [28] were provided for in-session reference. For the lifestyle block, “truth or myth” statements were shown on slides and participants responded using large double-sided placards (“myth” or “truth”) held up during the discussion. For the emotions block, participants used emotion cards and small envelopes to complete the “box of emotions” exercise. At the end of the session, each participant received a take-home brochure, in Catalan, containing key messages from the workshop.

### 3.6. Stage 6: Implementation

#### 3.6.1. Stage 6.1: Inclusion and Exclusion Criteria for Participants

The inclusion criteria were as follows: individuals aged 18 years or older residing in the Segrià region. Conversely, the exclusion criteria included individuals who faced a language barrier with either Catalan or Spanish.

#### 3.6.2. Stage 6.2: Mode of Delivery

A 2 h, in-person workshop format was selected for each municipality, combining a brief didactic segment with interactive, hands-on components and peer discussion. This mode supported facilitator control over fidelity, enabled rapid clarification of misconceptions, and leveraged community dynamics. Sessions were scheduled with municipal councils in accessible venues and were language-tailored (Catalan/Spanish).

#### 3.6.3. Stage 6.3: Implementation Plan and Fidelity Assurance

Implementation was coordinated by a university-based research group with input from two HE experts and logistical support from the Regional Council and municipal authorities. Facilitators completed a 2–3 h briefing covering scripts, sequencing, timing, and feedback techniques, followed by a mock session to calibrate delivery.

The session flow was standardized—introduction (~10 min), Activity 1: hand hygiene (~35 min), Activity 2: healthy habits and COVID-19 (~30 min), Activity 3: emotions (~45 min), and an integrated closing—while allowing minor time reallocations across activities based on group needs, without altering the sequence or core components.

Recruitment and logistics were managed in partnership with the different municipalities (posters, WhatsApp/e-Bando announcements, social media, and the town crier). Venue capacity set the group size, and scheduling was aligned with community availability. Governance included internal team meetings approximately every three weeks for process monitoring, material stock, and coordination with municipal councils.

#### 3.6.4. Stage 6.4: Development of the Intervention and Conducting of Formative Piloting

Materials were produced after internal usability checks, and the first two workshops (two municipalities) were treated as an opportunistic formative pilot. Refinements were made according to the facilitator’s session notes, immediate post-session debriefs, the post-session satisfaction/Net Promoter Score (NPS) questionnaire (with two open-ended items), and participants’ one-word descriptors. Adaptations were recorded in session notes and discussed during debriefs.

#### 3.6.5. Stage 6.5: Modifications

During early rollout, as mentioned above, the first two workshops were used as an opportunistic formative pilot. Two adjustments were made and then retained for the remainder of delivery. First, the hand hygiene ordering item in the pre/post questionnaire was simplified. The original version listed 10 individual stages to be ordered; this was consolidated to 7 grouped options and finally to 5 grouped options, to improve feasibility within a single session and to emphasize logical sequencing over memorization of granular sub-steps. This change concerns the evaluation instrument rather than the intervention content and is reported here for transparency. Second, within the emotions activity, facilitators reinforced verbal guidance by providing clearer instructions prior to card selection and sharing. This adjustment did not alter the materials, sequence or core components of the activity. No other modifications were made. The slides, brochure, “myth or truth” placards, and emotion cards remained unchanged. Likewise, the language used, examples provided, recruitment messages (managed by the municipalities), physical spaces and equipment, and consumables (such as gloves and paint) did not require any changes.

During the routine delivery, minor time reallocations were made at the facilitators’ discretion (e.g., extending the hand hygiene practice segment when more errors were observed). These adjustments were compensated for by slight reductions in other parts of the session and did not affect the core components. All decisions were documented in session notes and discussed during post-session debriefings.

### 3.7. Stages 7 and 8: Evaluation and Follow-Up

Although a quasi-experimental design with pre- and post-intervention observations was used in the example described, this article focuses on the methodological process rather than on statistical effectiveness outcomes.

#### 3.7.1. Stage 7.1: Data Collection and Evaluation Plan

Data collection focused on reach, acceptability, and delivery quality to support quality improvement and future effectiveness analyses. No effectiveness comparisons (pre–post or versus control) are reported in this manuscript. Instruments and timing were as follows:

- Attendance/Reach: participant counts per municipality; basic sociodemographic data (gender, age, education, and area of residence).
- Acceptability/Satisfaction: seven items (0–10) on clarity, usefulness, and relevance; overall satisfaction (0–10); two open questions; and NPS classification (promoters 9–10; passives 7–8; detractors 0–6).
- Fidelity: facilitator delivery checklists; session notes on deviations; and post-session debrief summaries (no formal adaptation framework or log was maintained).

The evaluation of the intervention was conducted at three specific time points:

- Before the intervention: a formative evaluation was carried out to assess the suitability of the intervention [6]. This included identifying population needs through a prior mixed-methods study involving an online questionnaire and two focus groups [10]. A pilot study was conducted in two municipalities of the Segrià region to test the activities and materials, and necessary improvements were incorporated.
- During the intervention: a process evaluation was performed based on the feedback from the HE workshops [6]. This included maintaining a participant and municipality attendance list, conducting follow-up meetings every three weeks with the research team, and administering the satisfaction questionnaire.
- After the intervention: an impact evaluation was conducted to determine whether the initial objectives were met [6]. This was assessed through pre- and post-intervention observations and questionnaires.

Regarding the pre- and post-intervention ad hoc questionnaires used for data collection, these included items designed to evaluate the content of the “Rethinking COVID-19: collective learning and experience” intervention. These items covered sociodemographic data (gender, age, education level, and area of residence), hand hygiene steps, healthy habits and COVID-19, and emotions (Table 2). Both questionnaires were anonymous, administrated in paper format, and self-completed by participants. To ensure anonymity, each participant was identified using an alphanumeric code.

#### 3.7.2. Stage 7.2: How Well (Planned)

To ensure consistent delivery across municipalities, this state was planned to assess fidelity at three levels: provider (facilitator preparedness), delivery (adherence to core components and timing), and receipt/enactment (participants’ engagement with practice and simple planning). In practical terms, the three facilitators (two nurses and one biotechnologist: two PhD and one pre-doctoral and two with specific training in HE) were responsible for fidelity in the sessions they delivered and for post-session debriefs with the rest of the team.

As an initial step, the first workshops were co-delivered by the three facilitators to calibrate roles, sequence and timing. Subsequent sessions were delivered by one facilitator, followed by a structured debrief with the other two to review session flow and resolve any issues, thereby maintaining alignment with the plan.

Before rollout, the core components were specified: (i) workshop introduction/overview, (ii) guided practice of hand hygiene, (iii) “truth or myth” activity, and (iv) “box of emotions” exercise. By contrast, adaptable elements included language (Catalan/Spanish) and minor time reallocation across activities according to group needs.

After each workshop, the delivering facilitator completed session notes (components covered, notable incidents, and approximate time distribution) and then held a brief debrief with the team. The team also met several times per month as needed to review notes and plan minor refinements. To avoid drift, materials were version-controlled (two versions in use) and stored in the university’s institutional OneDrive.

Finally, to check receipt/enactment, participants’ practice of handwashing was directly observable during the gloves-and-paint drill. In addition, the pre/post questionnaires contained items on the handwashing steps, enabling a basic procedural knowledge check (analyses of effectiveness are reported elsewhere).

#### 3.7.3. Stage 7.3: How Well (Actual)

In practice, all sessions covered the four core components as planned (introduction, guided handwashing practice, truth or myth activity, and “box of emotions” exercise). This consistent structure was maintained across venues and groups.

Where deviations occurred, they were minor and context-responsive. The only recurrent adjustment was allocating ~10 additional minutes to the handwashing practice when more errors were detected; this was compensated for by a slight reduction in another activity. No re-ordering of activities was required. A sink was not necessary because the practice of handwashing was performed with gloves and paint. Seating was adapted to room size and group numbers but did not affect delivery.

Judgements about fidelity were based on facilitator session notes and post-session debriefs. Pre/post questionnaires and the satisfaction form provided process information that supported minor refinements; these sources were not used to estimate effectiveness in this article.

With respect to receipt/enactment, all groups practiced the handwashing drill and brief action-planning elements (when prompted within activities) proceeded without notable exceptions.

## 4. Discussion

This article describes the development and implementation of a pragmatic, theory-based community HE intervention, designed to improve KAP related to COVID-19 prevention and management. To that end, the Eight-Stage Health Education Planning Instrument was applied as a structured, iterative framework guiding the process from situation analysis to evaluation and follow-up. Each stage introduced explicit decision points and deliverables, ensuring methodological consistency while allowing contextual flexibility.

This instrument builds upon established planning frameworks, but it differentiates itself by operationalizing the planning process through standardized stages and deliverables. To our knowledge, this article represents the first scientific effort to formally describe the Eight-Stage Health Education Planning Instrument and to illustrate its application from theory to practice in a community HE context.

The planning process was complemented by behavioral models (ASE model, TTM, and I-Change model) and the BCW. This model provided a solid theoretical foundation and enabled a deeper understanding of participants’ behaviors and motivations, thereby facilitating the design of more effective educational strategies and the promotion of health [12]. As with a previous study that also employed planning frameworks to guide intervention design [33], this systematic integration allowed the team to link behavioral determinants with specific techniques, ensuring that the intervention addressed not only knowledge gaps but also attitudes, motivation, and behavioral skills. By specifying and reporting the intervention using the TIDieR checklist, the authors aimed to ensure transparency and replicability for future research.

Although it is true that since the start of the pandemic, many interventions have been performed in the population in general [34,35], others have focused on specific groups such as adolescents [36,37], health degree students [38,39,40], or school children [41,42]. However, in most of the interventions, a single topic was addressed, such as hand hygiene [41], vaccination [35], mental health [37] or knowledge about COVID-19 [36]. In addition, given the need to rapidly reach the population, some of these interventions were carried out online, through webinars [38] or videos [34], which was also due to the pandemic situation and the restrictions imposed to avoid the propagation of the virus. Therefore, this intervention is notable for its comprehensive and personalized approach to the population, while the instrument is distinguished by integrating a complete planning framework with behavioral theory and by documenting its practical application step by step.

In line with previous research, theory- and behavior-based HE interventions have shown effectiveness across diverse populations and contexts, including adolescents, students, and community settings, when appropriately adapted to local needs [34,35,36,37,38,39,40,41,42]. These studies support the relevance of integrating behavioral determinants, stages of change, and structured planning frameworks, reinforcing the external validity of the present methodological approach.

Although the “Rethinking COVID-19” intervention was tailored to the Segrià region, the Eight-Stage Health Education Planning Instrument is inherently adaptable. Potential adaptations for other socio-cultural contexts may include adjustments to communication strategies (e.g., simplified language, culturally relevant messaging, and use of community mediators), educational materials (e.g., context-specific examples and multilingual resources), and delivery formats (e.g., modular sessions and digital or hybrid approaches). Importantly, these adaptations can be implemented without altering the core theoretical structure of the instrument, which remains grounded on behavioral theory and systematic planning.

Another fundamental aspect is the use of formative pilot workshops that further strengthened this responsiveness, as facilitator notes, participant feedback, and immediate debriefs were incorporated into iterative refinements. This process ensured that core components were preserved, while small, context-sensitive adjustments improved clarity and delivery. This approach is also supported by recent research on health interventions, which highlighted the value of pilot testing in improving feasibility and tailoring content to specific populations [43]. This flexible yet structured approach exemplifies how theoretical rigor and practical adaptability can coexist within community HE.

Lastly, one of the main strengths of the study is that it was designed considering the specific needs of the population. This approach increases the probability that the interventions will be efficient in the promotion of healthy behaviors and the reduction in health risks [39]. Despite this strength, the study also has limitations. The most important is the absence of a long-term follow-up, which prevents us from assessing whether improvements in KAP were sustained over time. Evaluations three to six months after participation would be necessary to determine whether the intervention leads to lasting behavioral change [12]. The limited sustainability was primarily due to the constrained duration of the project, which required focusing on the immediate implementation and assessment of the instrument. In addition, logistical challenges related to following up with the same participants over an extended period made long-term evaluation unfeasible. Furthermore, the workshop was tested in a specific community context; while the structured use of theory and transparent reporting enhance replicability, adaptations may be needed for other populations and cultural settings.

## 5. Conclusions

The Eight-Stage Health Education Planning Instrument provides a structured yet adaptable roadmap for the design, implementation, and evaluation of community HE programs. Its systematic stages, from situation analysis to sustainability, offer a practical framework for integrating theory, evidence, and community context in a coherent way.

Through its application to the “Rethinking COVID-19” intervention, this article demonstrates how the instrument can standardize planning while maintaining flexibility to address local constraints and emerging needs. The integration of behavioral theories and the clear differentiation between evaluation and follow-up further enhance its methodological robustness and potential for institutionalization.

This approach contributes to bridging the gap between theory and practice in HE planning, supporting transparent, replicable, and sustainable interventions. By applying and reporting the Eight-Stage Health Education Planning Instrument, this study provides a replicable example that can provide information for future methodological developments and guide the transition from pandemic-specific interventions to broader community preparedness strategies.

## Figures and Tables

**Table 1:** Conceptual and operational alignment between the Eight-Stage Health Education Planning Instrument, PRECEDE–PROCEED, and Intervention Mapping.

Eight-Stage Health Education Planning Instrument	PRECEDE–PROCEED	Intervention Mapping	Purpose
1–3 (situation analysis, needs and problem identification, prioritization)	PRECEDE 1–3	Step 1	Define priority gaps; target behaviors and determinants
4–5 (goals → activities and resources)	PRECEDE 4 → PROCEED 5	Steps 2–4	Tie targets to theories; translate to practical applications
6–8 (implementation, evaluation and follow-up)	PROCEED 5–8	Steps 4–6	Produce materials; plan implementation and fidelity; formative piloting

**Table 2:** Items and measurement instruments used in the “Rethinking COVID-19” intervention.

Item		Measurement Instrument
Sociodemographic	Gender	Questions created by the authors.
	Age	
	Level of education	
	Area of residence	
Hand hygiene		Handwashing checklist with soap and water proposed by the Generalitat de Catalunya, Catalan Health Service (2020) [28].
Healthy habits and COVID-19	Importance of doing physical exercise	Question adapted from the validated Spanish version of the Regicor short physical activity questionnaire for the adult population [29].
	Quality and adherence to the Mediterranean diet	Question adapted from a validated 14-item questionnaire of Mediterranean diet adherence [30].
	The relationship between sedentary lifestyle, electronic devices and sleep disorders	Question adapted from the validated Spanish version of the Regicor short physical activity questionnaire for the adult population [29].
	COVID-19 and information	Question adapted from the WHO questionnaire on COVID-19 [31].
	Beliefs about the vaccine against COVID-19	Question adapted from the WHO questionnaire on COVID-19 [31].
Emotions	Experienced emotions and coping measures	Questions adapted from European Spanish version of the Perceived Stress Scale [32].

## Data Availability

The raw data supporting the conclusions of this article will be made available by the authors on request.

## Abbreviations

The following abbreviations are used in this manuscript:

ASE	Attitude, Social Influence, and Efficacy
BCW	Behavior Change Wheel
HE	Health Education
KAP	Knowledge, Attitudes, and Practices
NPS	Net Promoter Score
TIDieR	Template for Intervention Description and Replication
TTM	Transtheoretical Model
WHO	World Health Organization

## Appendix A. TIDieR Checklist (Completed for “Rethinking COVID-19”)

**Table A1:** This checklist documents where each TIDieR item is described in the manuscript and how to access materials to support replication [25].

Item Number	Item	Location of Primary Paper (Page or Appendix Number)
	BRIEF NAME	
1.	Provide the name or a phrase that describes the intervention.	6
	WHY	
2.	Describe any rationale, theory, or goal of the elements essential to the intervention.	7–8
	WHAT	
3.	Materials: Describe any physical or informational materials used in the intervention, including those provided to participants or used in intervention delivery or in training of intervention providers. Provide information on where the materials can be accessed (e.g., online appendix, URL).	10
4.	Procedures: Describe each of the procedures, activities, and/or processes used in the intervention, including any enabling or support activities.	9–10
	WHO PROVIDED	
5.	For each category of intervention provider (e.g., psychologist, nursing assistant), describe their expertise, background and any specific training given.	10
	HOW	
6.	Describe the modes of delivery (e.g., face-to-face or by some other mechanism, such as internet or telephone) of the intervention and whether it was provided individually or in a group.	10
	WHERE	
7.	Describe the type(s) of location(s) where the intervention occurred, including any necessary infrastructure or relevant features.	10
	WHEN and HOW MUCH	
8.	Describe the number of times the intervention was delivered and over what period of time including the number of sessions, their schedule, and their duration, intensity or dose.	10
	TAILORING	
9.	If the intervention was planned to be personalized, titrated or adapted, then describe what, why, when, and how.	6–7
	MODIFICATIONS	
10.	If the intervention was modified during the course of the study, describe the changes (what, why, when, and how).	11
	HOW WELL	
11.	Planned: If intervention adherence or fidelity was assessed, describe how and by whom, and if any strategies were used to maintain or improve fidelity, describe them.	13
12.	Actual: If intervention adherence or fidelity was assessed, describe the extent to which the intervention was delivered as planned.	13
